# Establishing Reference Intervals for Fat-Soluble Vitamins in Healthy Chinese Adults: Study Protocol for a Cross-Sectional, Multicenter Study

**DOI:** 10.2196/80425

**Published:** 2026-02-17

**Authors:** Zhong Liu, Chenbing Liu, Nan Li, Chao Shen, Lihong Qiu, Di Sheng

**Affiliations:** 1 Department of Health Management Center, The First Affiliated Hospital of Zhejiang University School of Medicine, 79 Qingchun Rd, Hangzhou, Zhejiang Province, 310003, China, 86 13957104885

**Keywords:** fat-soluble vitamins, reference intervals, Chinese population, physical examination, multicenter

## Abstract

**Background:**

Fat-soluble vitamins (FSVs)—vitamins A, D, E, and K—are essential micronutrients involved in key physiological processes. Both deficiency and excess can influence nutritional assessment and disease risk. In China, clinical evaluation of FSVs often relies on reference intervals (RIs) derived from Western populations, and no large-scale study has comprehensively evaluated all 4 FSVs in healthy Chinese adults.

**Objective:**

The aim of this study is to establish population-specific RIs for vitamins A, D, E, and K in healthy Chinese adults, thereby supporting more accurate clinical assessment of vitamin status and the prevention of related diseases.

**Methods:**

This ongoing multicenter cross-sectional study aims to recruit 100,000 adults (≥18 y) from 20 hospitals across China. Participants undergo a standardized questionnaire, physical examination, and blood sample collection. FSV concentrations are quantified using liquid chromatography-tandem mass spectrometry. Group differences are assessed using the chi-square test or Fisher exact test. Nested ANOVA evaluates variation across subgroups. RIs will be established using parametric or nonparametric percentile methods following rigorous outlier removal to accurately determine the 2.5th and 97.5th percentile limits.

**Results:**

As of October 2025, 13,545 adults have been enrolled, and 1690 participants have met the inclusion criteria. Recruitment began on 1 July 2024 and is expected to conclude by 30 June 2026.

**Conclusions:**

This study will generate the first large-scale, population-specific RIs for FSVs in healthy Chinese adults. The findings are expected to be published in 2027 and will provide an important evidence base for clinical nutrition assessment and disease prevention in China.

## Introduction

Fat-soluble vitamins (FSVs), including A, D, E, and K, are micronutrients that play essential roles in maintaining human health. Unlike water-soluble vitamins, these nutrients are stored in the adipose tissues, liver, and muscles, making them prone to accumulation and potential toxicity [1]. Vitamin A is crucial for vision, immune function, and cellular communication [2]. Its deficiency can lead to impaired vision, particularly night blindness, and an increased risk of infections [3]. Vitamin D is well-known for regulating calcium homeostasis and bone metabolism. Deficiency in vitamin D can result in different pathologies such as diabetes, certain cancers, cardiovascular diseases, fertility, and many other conditions [4]. Vitamin E, primarily an antioxidant, is essential for nervous system development, growth, and immunity [5]. Vitamin E deficiency is associated with increased infection, anemia, nervous system abnormalities, and stunted growth [6]. Vitamin K is essential for blood coagulation and bone health, while insufficient vitamin K leads to higher risk of bone fracture and cardiovascular disease [7]. Because of these essential functions, maintaining appropriate levels of FSVs is crucial; both deficiency and excess can influence nutritional assessment and clinical decision-making.

Reference intervals (RIs) serve as the basis of laboratory testing and aid the physician in differentiating between the healthy and diseased patients. Standard methods for determining the RIs are to define and obtain a healthy population of at least 120 individuals and use nonparametric estimates of the 95% RI [8]. In China, the need for population-specific RIs is particularly pressing due to the country’s vast genetic diversity and unique environmental and lifestyle factors. Many clinical laboratories in China rely on guideline- or manufacturer-provided RIs, which may not fully reflect the Chinese population and can contribute to misclassification and inappropriate clinical decisions [9,10]. Therefore, developing and periodically updating RIs tailored to the Chinese population is crucial for effective health care delivery and public health management.

Research on FSVs often focuses on specific vitamins, particular subgroups, or limited regions, leading to a lack of comprehensive data tailored to the healthy Chinese adult population. For example, global research on vitamin D emphasizes widespread deficiency among diverse populations and its correlation with chronic diseases [11]. A recent study from a southwestern province reported RIs for vitamins A and E, but its relatively small sample size limits its generalizability to the broader Chinese population [12]. Previous studies on vitamin E status have focused on elderly cohorts [13], infants [14], or specific regional populations [12], and lack conclusive data [15], underscoring the need for broader research that reflects the overall healthy adult population. Similarly, vitamin K research often focuses on its deficiency in osteoporosis, cardiovascular contexts, or other related diseases, neglecting its broader nutritional status in populations [16-18]. Therefore, this gap is especially notable in China, as no large-scale study has analyzed all 4 FSVs in a healthy adult population. Nonetheless, current clinical practice often relies on RIs based on Western studies, which may not fully capture the nutritional needs of Chinese individuals. Factors such as dietary patterns, geographic diversity, and cultural practices can all influence FSV levels. Given the substantial differences in lifestyle and dietary patterns between Chinese and Western populations, establishing population-specific RIs is essential for accurate nutritional assessment and public health guidance.

To address these issues, this study presents a protocol for establishing population-specific RIs for FSVs in a healthy Chinese adult population. Using a cross-sectional study design, we will collect a representative sample of individuals across diverse regions and demographics to account for variations in lifestyle and environmental exposure. This protocol outlines a detailed methodology for conducting the study, including participant selection, laboratory methods for vitamin quantification, and statistical techniques for establishing the RIs. To the best of our knowledge, this is the first cross-sectional study to assess the RIs for FSVs in this specific population. These RIs will offer clinicians more accurate tools for evaluating vitamin status and supporting public health efforts, ultimately helping prevent diseases related to both deficiency and excess.

## Methods

### Study Design

**Figure 1.**
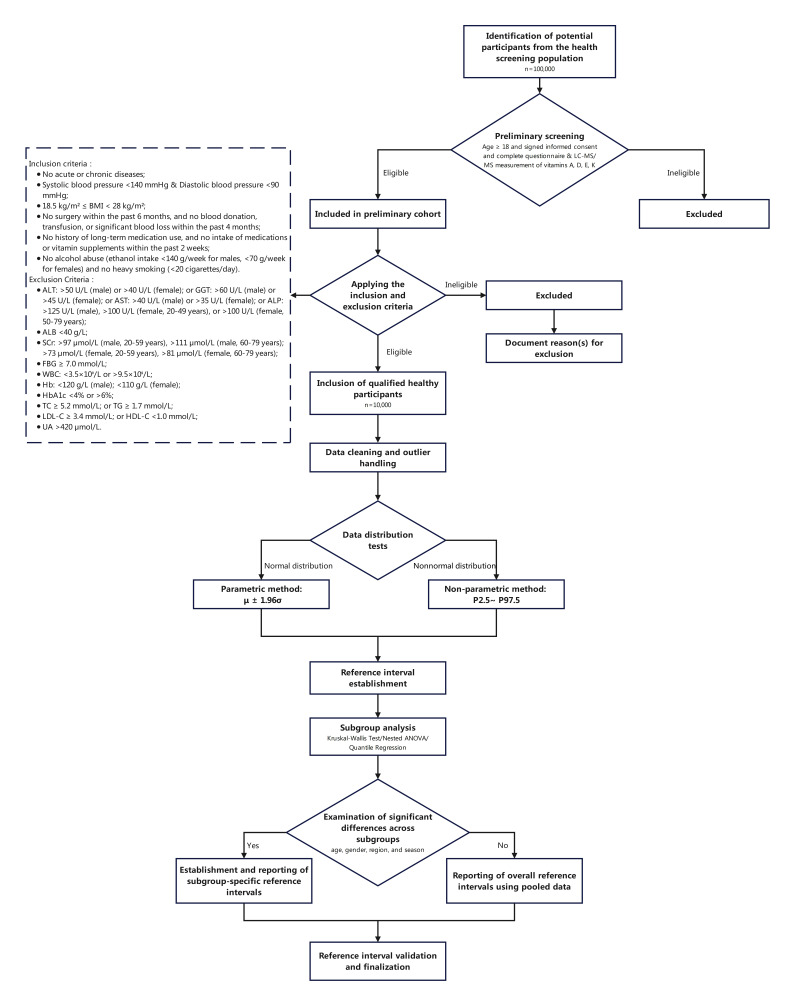
A flowchart of the study plan.

This is a multicenter, cross-sectional study. Participants will be recruited from 20 participating hospitals in China. The First Affiliated Hospital of Zhejiang University designed and initiated the study. All participating hospitals will be required to conduct the study in strict accordance with the established rules. Figure 1 presents a summary of the study process.

### Patient and Public Involvement

Neither participants nor the public were involved in the design, implementation, reporting, or dissemination planning of this research.

### Data Collection

Data collection consists of a standardized questionnaire and a comprehensive physical examination with laboratory testing. Participants are individuals undergoing health check-ups at participating hospital centers. Before the check-up begins, trained nurses confirm the selected examination package and invite participants to complete the questionnaire, which captures demographic characteristics, medical and family history, smoking and alcohol use, physical activity, and medication or supplement use (details in Table 1). Afterward, participants undergo standardized physical examinations, including measurements of height, weight, body composition, and blood pressure. Laboratory evaluations include a comprehensive metabolic panel, lipid panel, liver function tests, and FSVs level test. All test instruments are calibrated following the manufacturer’s guidelines. Blood samples are collected following an overnight fasting of 12 hours.

**Table 1.** The core components of the questionnaire.

Questionnaire outline	Description of content
Basic personal details	ID number, contact information, age, gender, place of residence, and ethnicity
Medical history	Severe cardiovascular and cerebrovascular diseases, severe liver and kidney disease, malignant tumors, acute and chronic infections, diabetes, and autoimmune diseases
Supplement use	Use of any supplements
Family medical history	Cardiovascular or cerebrovascular diseases, severe liver and kidney disease, malignant tumors, diabetes, and autoimmune diseases
Alcohol consumption	History of alcohol consumption, frequency, and type of alcohol consumed
Smoking	Number of cigarettes per day
Physical activity	Current and past participation in physical activities, weekly exercise duration, and intensity
Sleeping habit	Average sleeping hours, sleep quality, history of insomnia, or other sleep disorders

Laboratory results generated during the health check-up are automatically uploaded into the hospital information system and subsequently extracted for statistical analysis, with all data stored in a secure, access-controlled database. Since study initiation, the data collection workflow has proceeded smoothly, indicating high feasibility and acceptability of the study procedures within the health check-up setting. The inclusion and exclusion criteria are mentioned in Textbox 1.

Textbox 1. **Inclusion and exclusion criteria.**

The eligibility criteria for inclusion are as follows:

1. Age ≥18 years old, both male and female.
2. Undergo a health examination in participating hospitals.
3. Agree to participate in the study and sign the informed consent form.
4. BMI <28 kg/m^2^ and ≥18.5 kg/m^2^ [19].

The criteria for exclusion are as follows:

1. Admission to the hospital as an in-patient during the previous 4 weeks or surgery performed within 6 months.
2. Individuals receiving vitamin supplements or drugs that can affect FSV metabolism or absorption (bile acid sequestrants, statins, antimicrobials, antiepileptic drugs, corticosteroids, etc) [20].
3. Women who are pregnant or breastfeeding.
4. Alcohol consumption was recorded as grams of pure alcohol per week (g/week). Alcohol consumption >140 g/week for males and >70 g/week for females and smoking (>20 cigarettes/day).
5. Previous history of severe cardiovascular and cerebrovascular diseases, severe liver and kidney disease, malignant tumors, acute and chronic infections, diabetes, and autoimmune diseases.
6. Individuals with glomerular filtration rate, alanine transaminase, gamma-glutamyl transferase, albumin, fasting glucose, hemoglobin A1c, uric acid, total cholesterol, triglyceride, low-density lipoprotein cholesterol, high-density lipoprotein cholesterol, and platelet count values falling outside the common reference [21].
7. Subtract replicates for the same individual (only the first value was considered).

### Data Quality Control

To ensure reliability, consistency, and accuracy of the study data, a multilevel data quality control (QC) strategy will be implemented, covering questionnaire surveys, sample collection and processing, laboratory testing, and data management.

#### Questionnaire Data QC

##### Training and Standardization

All research staff involved in the survey will receive standardized training to ensure consistent interpretation and uniform administration of the questionnaire.

##### Recall Bias Verification

To assess and control recall bias, 100 participants will be randomly selected to complete the questionnaire a second time, approximately 4 weeks after the initial administration. The second questionnaire will be identical to the first. To ensure independence, the follow-up survey will be conducted either through a self-administered electronic questionnaire or a telephone interview conducted by a different research assistant. Consistency of key variables (eg, supplement use and smoking or alcohol history) will be evaluated using the kappa coefficient or intraclass correlation coefficient (ICC). Kappa or ICC values >0.75 will be interpreted as good reliability, 0.60‐0.75 as moderate, and <0.60 as poor.

##### Logical Checks

During data entry, logical skip patterns and range checks will be implemented to automatically flag inconsistent or contradictory responses, which will then be reviewed and verified by research staff.

##### Electronic Data Collection

Electronic questionnaire systems will be used whenever possible to minimize manual data entry errors.

### Standardization of Sample Collection, Transport, and Storage

#### Unified Standard Operating Procedures

All participating centers will follow the same standard operating procedures for sample collection, processing, storage, and transport.

#### Sample Labeling and Tracking

A barcode-based system will be used to label and track samples, ensuring full traceability from collection to analysis.

#### Transport Monitoring

Temperature monitoring devices will be used to record temperature conditions during transport, ensuring that samples remain within acceptable ranges.

### Laboratory Testing QC

#### Methodological Standardization

All participating laboratories will use the same liquid chromatography-tandem mass spectrometry (LC-MS/MS) platform, reagent brands, and testing protocols.

#### Internal QC

Each analytical batch will include commercial QC materials at low, medium, and high concentrations, as well as pooled human serum controls. Results must meet predefined acceptance criteria within ±2 SD.

#### External Quality Assessment

Laboratories will regularly participate in national or international proficiency testing programs for FSVs to ensure analytical accuracy and interlaboratory comparability.

#### Central Laboratory Retesting

Each month, 2% (2/100) of samples from each participating center will be randomly selected and sent to the central laboratory for repeat testing. Intercenter consistency will be assessed using Bland-Altman analysis or ICCs, with corrective actions implemented if necessary.

### Data Management QC

#### Dual Independent Data Entry

Paper-based questionnaire data will be entered electronically by 2 independent operators. Any discrepancies will be resolved by a third reviewer with reference to the original records.

#### Data Cleaning and Review

Regular data cleaning procedures will be performed, including outlier detection, identification of missing values, and logical consistency checks.

#### Preanalysis Data Verification

Before statistical analysis, a statistician will review the final dataset for completeness, distribution characteristics, and potential sources of biases.

### Documentation of QC Activities

All QC procedures, results, and records related to exception handling will be documented in detail and archived to ensure methodological transparency and to support future audits and manuscript preparation.

### FSVs Quantification

#### LC-MS/MS-Based Quantification of FSVs

This study will employ LC-MS/MS to quantitatively analyze FSVs in human blood samples. LC-MS/MS is widely utilized for the quantitative analysis of small molecules in complex biological matrices, such as blood, plasma, and urine, due to its high sensitivity and specificity [22,23]. For vitamin A analysis, recent studies have developed and validated LC-MS/MS methods for the simultaneous quantification of retinol and its metabolites in human serum, demonstrating high sensitivity and specificity suitable for clinical applications [24]. In the analysis of vitamin E, validated LC-MS/MS assays allow simultaneous determination of tocopherols and their oxidative metabolites in plasma and serum, providing excellent sensitivity, minimal sample preparation, and high-throughput capabilities [25-27]. For vitamin D quantification, LC-MS/MS has been widely applied to accurately measure serum 25-hydroxyvitamin D_₂_ and 25-hydroxyvitamin D_₃_ concentrations, offering superior specificity and precision compared to traditional immunoassays [28]. Furthermore, recent advancements, such as the development of derivatization techniques, have enabled the simultaneous quantification of multiple vitamin D metabolites with enhanced sensitivity, particularly in patients with diabetes and hyperlipidemia [29]. Additionally, recent LC-MS/MS protocols have been developed for the determination of different vitamin K forms in human plasma, which are essential for clinical evaluation [30]. In this study, the analytical method is based on established protocols with minor modifications to support high-throughput analysis. Briefly, samples will undergo protein precipitation followed by liquid-liquid extraction using organic solvents. Chromatographic separation will be performed on a reversed-phase C18 column, and detection will be carried out in multiple reaction monitoring mode. Quantification will be achieved using calibration curves constructed with isotope-labeled internal standards for each analyte, allowing correction for matrix effects and variability in extraction recovery.

#### Method Validation Parameters

Prior to study initiation, the LC-MS/MS method was fully validated in the central laboratory in accordance with International Council for Harmonisation (ICH) M10 guidelines [31] to ensure reliability. The key validation parameters are summarized below.

##### Precision

Intraday precision (repeatability) and interday precision (intermediate precision) were assessed by analyzing QC samples at 3 concentration levels (low, medium, and high) across multiple runs. Coefficients of variation were ≤8% for intraday precision and ≤12% for interday precision for all analytes.

##### Accuracy

Accuracy was assessed using spike-and-recovery experiments and by analyzing certified reference materials, where available. Mean recovery rates ranged from 92% to 108% across the validated concentration ranges for all vitamins.

##### Linearity

The method demonstrated linearity over clinically relevant concentration ranges (eg, vitamin D: 5‐250 nmol/L) with ICCs (R²)>0.995 for all calibration curves.

##### Limits of Detection and Limits of Quantification

The limits of detection and limits of quantification (LOQ) were determined based on signal-to-noise ratios of 3:1 and 10:1, respectively. The LOQs were established below the lower limit of the expected physiological ranges for each vitamin.

##### Specificity and Selectivity

No significant interference from endogenous compounds or commonly used medications was observed at the retention times of the target analytes or their corresponding internal standards.

### Intersite Harmonization and Quality Assurance

A rigorous, multilayered strategy is implemented to ensure the comparability of results across all 20 participating hospitals.

#### Standardization of Preanalytical and Analytical Procedures

All sites adhere to identical, detailed standard operating procedures for sample collection (fasting status, tube type), processing (centrifugation speed and duration), storage (−80 °C), and shipment (on dry ice with continuous temperature monitoring).

#### Unified Analytical Platform and QC Materials

All participating laboratories use the same model of LC-MS/MS instrument, identical reagent suppliers, and a standardized analytical protocol. A common batch of commercial, multilevel QC materials (low, medium, and high) is provided to each site. Additionally, a centrally prepared pooled human serum QC sample is distributed monthly.

#### Mandatory QC Runs

Each analytical batch must include all 3 levels of QC samples. A batch is accepted only if all QC results fall within predefined limits (±2 SD of the established mean).

#### External Quality Assessment

All laboratories are required to participate and successfully complete relevant national or international external quality assessment programs on a biannual basis.

#### Central Laboratory Retesting Program

On a monthly basis, 2% of samples from each center, randomly selected, are shipped to the central coordinating laboratory for blind reanalysis. Intercenter agreement is assessed using Bland-Altman analysis and ICC.

#### Data Harmonization and Corrective Action

An ICC ≥0.90 is considered indicative of excellent agreement. If systematic bias is identified, an investigation is triggered, and corrective actions (such as instrument recalibration or personnel retraining) are implemented before the site resumes sample analysis. This process ensures continuous longitudinal harmonization across the study network.

### Sample Size Estimation

According to the Chinese Health Industry Standard for Establishing RIs for Clinical Laboratory Test Items [32], to ensure the reliability of the results, this study plans to recruit 100,000 participants to establish RIs for FSVs A, D, E, and K in the adult Chinese population. The sample size estimation for establishing RIs was conducted based on a multicenter, cross-sectional study design. The goal was to recruit a total of 100,000 adult individuals from the physical examination cohorts of 20 participating hospitals across China. Based on existing literature and pilot data, it was estimated that approximately 10% of the screened population would meet the predefined health criteria for RI establishment. Therefore, the final analytical cohort was projected to include approximately 10,000 eligible and healthy adults.

The stratification strategy prioritized core biological variables. Participants were stratified by sex (2 strata) and age (5 strata: 18‐30, 30‐40, 40‐50, 50‐60, and >60 y), resulting in 10 primary strata. With an expected sample of 10,000 healthy individuals, the average sample size per stratum would be 1000, which substantially exceeds the Clinical and Laboratory Standards Institute (CLSI) EP28-A3 recommendation of at least 120 subjects per stratum for nonparametric RI estimation [33]. This ensures sufficient power and precision for estimating percentile-based RIs.

Environmental factors, including geographical region (North/South) and season (spring, summer, autumn, or winter), were not included as independent stratification variables during initial sample size calculation to avoid excessive fragmentation and insufficient sample sizes within multiple substrata. Instead, these variables will be evaluated as covariates during statistical analysis (eg, using analysis of covariance or quantile regression). If region or season demonstrates a statistically and clinically meaningful influence on vitamin concentrations, separate RIs will be considered. In that scenario, post hoc stratification (eg, region×sex×age=20 strata) would still result in an average of approximately 500 individuals per stratum, exceeding the CLSI minimum requirement.

This sampling framework ensures that the study is adequately powered to establish precise sex- and age-specific RIs for the Chinese population, while maintaining sufficient statistical robustness to explore potential effects of region and season.

### Statistical Data Analysis

#### Descriptive and Comparative Analyses

All statistical analyses will be performed using SPSS (version 28.0.1.1), GraphPad Prism (version 9.5.0), and R software (version 4.2.1). Continuous variables will be assessed for normality using the Kolmogorov-Smirnov test and visual inspection of Q-Q plots. Normally distributed data will be presented as mean (SD) and nonnormally distributed data as median (IQR). Categorical variables will be expressed as frequencies and percentages. Group comparisons for continuous variables will be conducted using independent-samples *t* tests for normally distributed data or Mann-Whitney *U* tests for nonnormally distributed data. For comparisons involving more than 2 groups, one-way ANOVA or Kruskal-Wallis tests will be applied, as appropriate. Comparisons of categorical variables will be performed using the chi-square test or Fisher exact test. To address multiple testing in planned subgroup analyses (eg, by age strata, region, and season), the false discovery rate will be controlled using the Benjamini-Hochberg procedure where applicable, with a significance threshold of *P*<.05.

#### RI Estimation Procedure

##### Overview

The establishment of RIs will follow a standardized, multistep process. A conservative consensus approach will be applied using 3 independent methods—Tukey boxplot method (1.5× IQR rule), the standard *z* score (|*z*|>3), and the modified *z* score (|M|>3.5). A data point will be excluded only if identified as an outlier by at least 2 of these 3 methods.

##### Distribution Assessment and Method Selection

For each vitamin within each predefined or partitioned subgroup, normality of the cleaned data will be reassessed. If the data follow a normal distribution, the central 95% RI will be calculated using a parametric approach (mean ±1.96× SD). If the data are nonnormally distributed, a nonparametric approach will be used, with the 2.5th and 97.5th percentiles directly defining the lower and upper reference limits.

##### CI Estimation for Reference Limits

To assess the precision of the estimated reference limits, 90% CIs will be calculated for both the lower (2.5th percentile) and upper (97.5th percentile) limits. For nonparametric estimates, bootstrap resampling with 1000 iterations will be used to generate percentile-based CIs. For parametric estimates, standard formulas based on the standard errors of the mean and SD will be applied. Reporting these 90% CIs provides an important measure of the reliability of the established RIs.

##### Rounding and Final Presentation

The final reference limits and their corresponding CIs will be rounded according to clinically relevant units (eg, to the nearest 0.1 nmol/L for vitamin D) to produce the final, publishable RIs.

### Subgroup Analysis

To determine whether separate RIs should be established for different subgroups, we will systematically evaluate the influence of 4 key factors: age, sex, geographic region, and season. The analysis will proceed in 2 sequential stages.

#### Stage 1*—*Assessment of Primary Stratification Factors (Age and Sex)

As age and sex are predefined stratification variables in the sampling design, we will first assess the necessity of partitioning RIs based on these factors. Vitamin concentrations will be compared across 5 age strata (18‐30, 30‐40, 40‐50, 50‐60, and >60 y) and between sexes using the Kruskal-Wallis test or ANOVA, as appropriate. If statistically significant differences (*P*<.05) with a standardized effect size greater than 0.30 are observed, age- and/or sex-specific RIs will be established.

#### Stage 2—Exploration of Environmental and Contextual Factors (Region and Season)

For factors not included in the initial stratification—geographic region (North vs South) and season (spring, summer, autumn, and winter)—a nested modeling approach will be used to evaluate their independent effects after accounting for age and sex. A nested ANOVA model will be constructed with FSV concentration as the dependent variable, age group and sex as fixed effects, and study center treated as a random effect nested within geographic region. A separate model will be used to assess the effect of season. If region or season explains a statistically significant proportion of variance (*P*<.05), its clinical relevance will be further evaluated by calculating age- and sex-adjusted standardized differences between groups.

The decision to establish separate RIs for region or season will follow a predefined 2-step criterion: (1) statistical significance (*P*<.05) and (2) clinical relevance, defined as a standardized difference greater than 0.30, indicating a small-to-moderate effect size.

#### Quantile Regression for RI Estimation

For any subgroup requiring separate RIs, the 2.5th and 97.5th percentiles will be estimated using quantile regression. This approach enables direct estimation of reference limits while adjusting for relevant covariates (eg, age and sex when establishing region-specific RIs), thereby improving the stability and precision of the estimates, even in subgroups with relatively smaller sample sizes.

#### Robustness Assurance

The study is adequately powered to support this level of partitioning. The planned sample size ensures that, even if RIs are stratified simultaneously by age, sex, region, and season, each resulting subgroup will include approximately 500 individuals, which exceeds the CLSI EP28-A3c minimum requirement of 120. If any partitioned subgroup fails to meet the predefined sample size threshold or does not satisfy the clinical relevance criterion (standardized difference ≤0.30), it will be merged with the most adjacent or clinically relevant group (eg, neighboring age strata or combined seasons) to preserve statistical robustness and clinical use. For all reported RIs, 90% CIs for the reference limits will be provided to ensure transparency regarding estimate precision.

### Ethical Considerations

This study complies with the Declaration of Helsinki and obtained ethical approval from the Clinical Research Ethics Committee of the First Affiliated Hospital, Zhejiang University School of Medicine (2024‐0728). Written informed consent will be obtained from all participants prior to enrollment. Participants’ privacy and confidentiality will be strictly protected; all personal data will be deidentified, securely stored, and be accessible only to authorized research personnel. This is an observational study and does not involve any treatment; therefore, participants will not receive any financial compensation. The findings of this study will be disseminated through conference presentations and peer-reviewed publications. Findings will be disseminated through publication in a peer-reviewed journal and presentations at conferences.

### Future Plans

On completion of subject recruitment, we will proceed with the systematic collection of relevant data, including (1) standardized questionnaires, (2) comprehensive physical examinations, and (3) blood samples for laboratory analysis. RIs will be established using parametric methods for normally distributed data and nonparametric methods otherwise, with values appropriately rounded. Nested ANOVA will be applied to assess the need for partitioning by gender, age, season, and region; groups without significant differences will be combined. To address potential sampling bias arising from the hospital-based health check-up population, future studies will prioritize validating the established RIs in external cohorts from more diverse socioeconomic and rural populations. Additionally, we plan to conduct longitudinal follow-up studies to track changes in FSV levels over time and examine their relationships with clinical outcomes.

## Results

As of October 2025, 13,545 adults have been enrolled, and 1690 participants have met the inclusion criteria. Recruitment began on 1 July 2024 and is expected to conclude by 30 June 2026. Data analysis is scheduled to begin on July 2026 and to complete by no later than December 2026. The projected end date of this study is December 2026, and results are expected to be published in May 2027.

## Discussion

This multicenter, cross-sectional study is designed to establish population-specific RIs for FSVs in a healthy Chinese adult population. Prior research on FSVs in China has been limited in scope and population coverage. Existing studies have typically focused on specific subgroups rather than establishing comprehensive national RIs: Yin et al [13] reported RIs for vitamins A and E in Chinese older adults ≥64 years, based on LC-MS/MS measurements; Nie et al [34] established vitamin K–related reference values in healthy women of childbearing age; and Yu et al [35] examined seasonal and age-specific vitamin D RIs in children. Although these studies contribute foundational data, they are constrained by age range, population specificity, or single-center design. In contrast, Western research has begun generating broader adult reference datasets. In European adults, Rigo-Bonnin et al [36] derived RIs for vitamins A and E using indirect data-mining approaches from over 2000 samples, and Caballero et al [37] later verified updated RIs using a direct approach in adults aged 18‐65 years. For vitamin D, population-based surveillance in Germany showed mean 25(OH)D levels around 45.6 nmol/L and a high prevalence of insufficient status [38]. No study has established comprehensive RIs across multiple FSVs in a large, diverse adult Chinese population, highlighting the need and significance of the current multicenter study. The RIs derived from this study are expected to serve as national laboratory benchmarks for healthy adults. The findings will provide valuable insights for clinicians to improve the assessment and management of FSVs and their related diseases.

The generalizability of the established RIs requires careful consideration because FSV concentrations are influenced by multiple population-level factors that vary across China. Dietary patterns, sunlight exposure, supplement use, and socioeconomic conditions differ substantially between urban and rural regions, and these differences may shift baseline vitamin distributions in ways not fully captured by our predominantly urban, health-check cohort. As a result, the current RIs may best reflect individuals with relatively stable health status and higher health awareness, rather than the broader nutritional and environmental diversity of the national population. In settings involving rural or nutritionally at-risk populations, these RIs should be interpreted with consideration of population-specific differences. Future validation in geographically and socioeconomically diverse cohorts will be essential to ensure that these RIs accurately represent the full spectrum of biological variability across the Chinese adult population.

The study has several strengths. First, the large-scale, multicenter design ensures that the sample is representative of the diverse demographic and geographic characteristics of China. This will allow the study to capture the regional variability in aging, lifestyle, and environmental exposures that influence FSV levels. Second, the standardized data collection process, which includes detailed questionnaires, physical examinations, and calibrated laboratory instruments, ensures the reliability and consistency of the collected data. Third, the inclusion of comprehensive physical and biochemical assessments, such as metabolic panels, lipid panels, and liver function tests, provides a comprehensive dataset for evaluating the factors that may influence FSV levels. Despite these strengths, the study has several limitations. First, this study uses a cross-sectional design with a single time-point measurement of FSV levels. Although nested ANOVA is applied to assess whether seasonal or regional partitioning of RIs is warranted, the design cannot capture within-individual variation in FSV levels related to seasonal dietary patterns, lifestyle fluctuations, or metabolic adaptation. As a result, the derived RIs may reflect average population distributions rather than the full physiologic variability experienced across the year. Second, the reliance on self-reported data for the questionnaire components introduces the potential for recall bias, particularly for lifestyle-related variables that influence vitamin status. A validation or reliability check was not performed, thus misclassification may have occurred and could affect subgroup comparisons. Third, our strict exclusion criteria likely yielded a “super healthy” cohort, potentially narrowing the estimated RIs compared with those from a more heterogeneous population. Therefore, caution is needed when applying these RIs in general clinical practice, and validation in more diverse cohorts is warranted. Finally, the study population consists of individuals undergoing routine health check-ups in economically developed urban regions. These individuals generally have better access to health care and may maintain healthier nutritional status, which may limit the generalizability of the derived RIs to adults from rural or lower-income areas. Nevertheless, this cohort reflects the population most likely to receive preventive laboratory testing in current clinical practice, making the resulting RIs highly relevant for routine use. Future comparative research involving more diverse geographic and socioeconomic groups, including relatively impoverished areas, is needed to validate the findings and enhance the generalizability.

This study holds critical importance for public health and clinical practice in China. Existing RIs for FSVs are mostly based on data from Western populations, which may not fully reflect the dietary habits, genetic traits, or environmental exposures of Chinese individuals. By establishing population-specific RIs, this research will improve the diagnostic accuracy of FSV assessments, enabling clinicians to better identify and manage both deficiencies and toxicities. Furthermore, the comprehensive data collected in this study will support the development of evidence-based public health strategies to prevent malnutrition-related health conditions. These findings are expected to contribute significantly to advancing precision nutrition and improving health outcomes for the Chinese population.

## Abbreviations

CLSI: Clinical and Laboratory Standards Institute
FSV: fat-soluble vitamin
ICC: intraclass correlation coefficient
LC-MS/MS: liquid chromatography-tandem mass spectrometry
LOQ: limits of quantification
QC: quality control
RI: reference interval
